# User Engagement Among Diverse Adults in a 12-Month Text Message–Delivered Diabetes Support Intervention: Results from a Randomized Controlled Trial

**DOI:** 10.2196/17534

**Published:** 2020-07-21

**Authors:** Lyndsay A Nelson, Andrew Spieker, Robert Greevy, Lauren M LeStourgeon, Kenneth A Wallston, Lindsay S Mayberry

**Affiliations:** 1 Department of Medicine Vanderbilt University Medical Center Nashville, TN United States; 2 Center for Health Behavior and Health Education Vanderbilt University Medical Center Nashville, TN United States; 3 Department of Biostatistics Vanderbilt University Medical Center Nashville, TN United States; 4 Institute for Medicine and Public Health Vanderbilt University Medical Center Nashville, TN United States; 5 Center for Diabetes Translation Research Vanderbilt University Medical Center Nashville, TN United States; 6 Department of Biomedical Informatics Vanderbilt University Medical Center Nashville, TN United States

**Keywords:** engagement, text messaging, mobile health, mHealth, mobile phone, technology, diabetes mellitus, type 2, self-management, self-care, medication adherence

## Abstract

**Background:**

Text message–delivered interventions are a feasible and scalable approach for improving chronic disease self-care and reducing health disparities; however, information on long-term user engagement with these interventions is limited.

**Objective:**

The aim of this study is to examine user engagement in a 12-month text message–delivered intervention supporting diabetes self-care, called REACH (Rapid Education/Encouragement And Communications for Health), among racially and socioeconomically diverse patients with type 2 diabetes (T2D). We explored time trends in engagement, associations between patient characteristics and engagement, and whether the addition of a human component or allowing patients to change their text frequency affected engagement. Qualitative data informed patients’ subjective experience of their engagement.

**Methods:**

We recruited patients with T2D for a randomized trial evaluating mobile phone support relative to enhanced treatment as usual. This analysis was limited to participants assigned to the intervention. Participants completed a survey and hemoglobin A1c (HbA1c) test and received REACH text messages, including self-care promotion texts, interactive texts asking about medication adherence, and adherence feedback texts. For the first 6 months, texts were sent daily, and half of the participants also received monthly phone coaching. After 6 months, coaching stopped, and participants had the option to receive fewer texts for the subsequent 6 months. We defined engagement via responses to the interactive texts and responses to a follow-up interview. We used regression models to analyze associations with response rate and thematic and structural analysis to understand participants’ reasons for responding to the texts and their preferred text frequency.

**Results:**

The participants were, on average, aged 55.8 (SD 9.8) years, 55.2% (137/248) female, and 52.0% (129/248) non-White; 40.7% (101/248) had ≤ a high school education, and 40.7% (101/248) had an annual household income <US $25,000. The median response rate to interactive texts was 91% (IQR 75%-97%) over 12 months. Engagement gradually declined throughout the intervention but remained high. Engagement did not differ by age, gender, education, income, diabetes duration, insulin status, health literacy, or numeracy. Black race and worse baseline medication adherence and HbA1c were each associated with lower engagement, although the effects were small. Nearly half of the participants chose to continue receiving daily texts for the last 6 months of the intervention. Participants who continued daily text messages said they wanted to continue experiencing benefits to their health, whereas those who chose fewer texts said that the daily texts had helped them create routines and they no longer needed them as often. Engagement was not impacted by receiving coaching or by participants’ chosen text frequency.

**Conclusions:**

Well-designed interactive text messages can engage diverse patients in a self-care intervention for at least 1 year. Variation in and reasons for frequency preference suggest that offering a frequency choice may be important to users’ engagement.

## Introduction

### Background

For many patients with chronic conditions, including type 2 diabetes (T2D), consistent, daily self-care (eg, taking medications, eating healthy, and exercising) is critical to avoid adverse health outcomes. However, patients with T2D face many barriers to self-care, including a lack of diabetes-related education and information, social barriers (eg, lack of motivation and caregiver stress), and difficulties with finances and transportation [1]. Racial/ethnic minorities and patients with low socioeconomic status (SES) tend to experience more barriers, which leads to worse self-care and health outcomes compared with non-Hispanic white patients and those with a high SES [2-5]. Text message–delivered interventions may offer an opportune pathway to extend self-care support to the hardest to reach and most vulnerable populations. More than 96% of adults in the United States use cell phones and find them to be essential for daily functioning [6,7]. Although smartphone ownership is growing and the digital divide has narrowed, individuals who have lower incomes, have less education, or live in rural areas are still less likely to have internet access or to own a smartphone [6,8,9]. Text messaging does not require internet access and is the most popular cell phone activity among all mobile phone users [10]. Evidence is growing for the efficacy of text message–delivered interventions to improve self-care for T2D and other chronic conditions [11-13]; however, there is a paucity of research examining how users engage with these interventions, particularly in the long term [14,15].

User engagement is essential to the ultimate success of any intervention. If target users are not attending to the intervention or lose interest quickly, potential effects may be attenuated or nullified. Approaches to measuring user engagement with technology-delivered interventions vary considerably across studies, making it a challenge to identify patterns and generate predictions. To help address this issue, Perski et al [16] synthesized past work on engagement with digital behavior change interventions to develop an integrative definition of engagement. They proposed engagement to be a multidimensional construct defined by both the extent of usage (often measured objectively) and a subjective experience characterized by attention, interest, and affect (often captured through qualitative data such as interviews) [16]. In many text message–delivered intervention studies, engagement is either not reported at all or reported using only a single metric (eg, average text message response rate) [15], which limits understanding of why participants were engaged, how engagement may change throughout an intervention, and factors contributing to engagement. Furthermore, among the studies reporting on engagement, intervention duration is typically 6 months or less [15,17] and, therefore, not representative of how users might engage in a longer experience. In these studies, text message response rates vary widely, but are often low, with some studies reporting rates as low as 17% [15,18,19]. Variability is likely due to variation in user attributes (eg, health literacy status and diabetes self-efficacy) and/or intervention characteristics across studies, but associations (or lack thereof) are often not reported [15]. In the few studies reporting associations, which span texting, interactive voice response, and internet-based interventions, patient characteristics such as older age [20], being nonwhite [20,21], and lower health literacy [20,22,23] have been linked to lower engagement.

Closely examining the features of the intervention may inform the conditions under which participants are more likely to engage. In text message interventions, specifically, the ideal frequency (ie, dose) of sending text messages to sustain engagement is unclear [12]. Suffoletto [24] referred to this as the Goldilocks problem: sending too few texts may not produce a strong enough effect, whereas sending too many texts may lead to participant fatigue and burnout. Furthermore, text messaging interventions often include additional modalities for delivering content, such as a human support component [25]. However, few studies have compared engagement between a condition receiving only automated text messages and a condition receiving texts in conjunction with human support.

Exploring engagement in long-term text messaging interventions is a critical step in understanding whether text messages are a viable option for improving and sustaining health outcomes. Identifying the determinants of intervention engagement can inform the design of interventions to optimize engagement [12,26]. Studies reporting on engagement with text messaging interventions tend to be short term, based on small sample sizes, and involve predominantly non-Hispanic white patients, limiting the understanding of long-term, generalizable results [17]. Important gaps in knowledge must be addressed to enhance the expanding use of text messaging technology to support chronic disease self-care in vulnerable groups at the greatest risk for worse outcomes.

### Objectives

We used mixed methods to examine user engagement in a 12-month text message–delivered intervention designed to support diabetes self-care, called REACH (Rapid Education/Encouragement And Communications for Health) [27,28]. We sought to explore how engagement changed over the course of the intervention, patient characteristics associated with engagement, whether the addition of a human component or letting participants choose their text frequency affected engagement, and patients’ reasons for their preferred text message frequency.

## Methods

### Study Design and Eligibility

This research was conducted as part of a larger randomized controlled trial (RCT), evaluating the effects of mobile phone–based support on diabetes self-care and hemoglobin A_1c_ (HbA_1c_) [27]. The trial and intervention details have been previously described in our development and protocol papers [27-29]. For this study, we analyzed data for participants who were randomly assigned to the intervention.

We recruited patients from federally qualified health centers (FQHCs) and Vanderbilt University Medical Center (VUMC) primary care clinics in and around Nashville, Tennessee. Eligible patients were aged ≥18 years, diagnosed with T2D, prescribed a daily diabetes medication, and responsible for administering their diabetes medications. In addition, patients were required to own a cell phone with text messaging capability and speak and read in English. We excluded participants whose most recent HbA_1c_ value within 12 months was <6.8% and who had auditory limitations or an inability to orally communicate, as determined by trained research assistants (RAs). We also excluded patients who failed a brief cognitive screener [30] to help ensure the accuracy of the measures and data integrity. Finally, due to the requirements of the intervention, we excluded patients who were unable to receive, read, or send text messages after a demonstration by an RA.

### Procedure

The Vanderbilt University institutional review board approved all study procedures. Interested and eligible patients completed informed consent, a baseline survey, and an HbA_1c_ test. RAs collected additional information from the participants’ electronic health record. RAs entered participants’ data into Research Electronic Data Capture (REDCap) [31]. Participants’ relevant survey responses were transferred from REDCap to a digital health platform called MEMOTEXT, via an automated application programming interface. MEMOTEXT used participant information to tailor, schedule, and send text messages to participants. When participants completed follow-up assessments at 3 and 6 months, updated patient information was used to retailor the text message content. When participants completed their 6-month assessment, they were given the option to continue receiving daily text messages or to receive fewer text messages, for the remaining 6 months of the intervention (described in *The Intervention* below). Participants could earn a total of US $210 for completing all study measures (ie, through 15 months for the larger RCT), with the payment schedule increasing for longer-term follow-ups.

We used strategic purposeful sampling to invite a subset of intervention participants to complete a follow-up interview after they finished their participation in the trial. Interviews were designed to assess perceptions of the intervention and understand dose choice and potential for implementation. Factors that informed our sampling approach included assigned condition, age, gender, race, education, income, clinic site, whether they owned a basic phone or smartphone, and their chosen text frequency. Interviews were conducted either in person or by phone and took approximately 20 min to complete (range 11-40 min). All interviews were audio recorded and transcribed verbatim. Participants were paid an additional US $40 for completing the interview. Herein, we report specifically on the participants’ comments about responding to text messages and reasons for their text message frequency choice after 6 months of receiving daily texts.

### The Intervention

All participants received self-care promotion text messages. Each message was either tailored to address user-specific barriers to medication adherence based on the Information-Motivation-Behavioral Skills model [32,33] or addressed other self-care behaviors (ie, diet, exercise, and self-monitoring of blood glucose). Participants chose a window of time to receive this text (eg, between 3 PM and 6 PM). Participants also received interactive text messages asking about medication adherence. We scheduled these to be sent after participants took their last dose of diabetes medication, but before their bedtime, to allow time to respond. At the end of each week, participants received an adherence feedback message based on their responses to the interactive text; feedback included an encouraging statement tailored to whether the participant’s adherence improved, stayed the same, or declined relative to the week prior.

**Figure 1:**
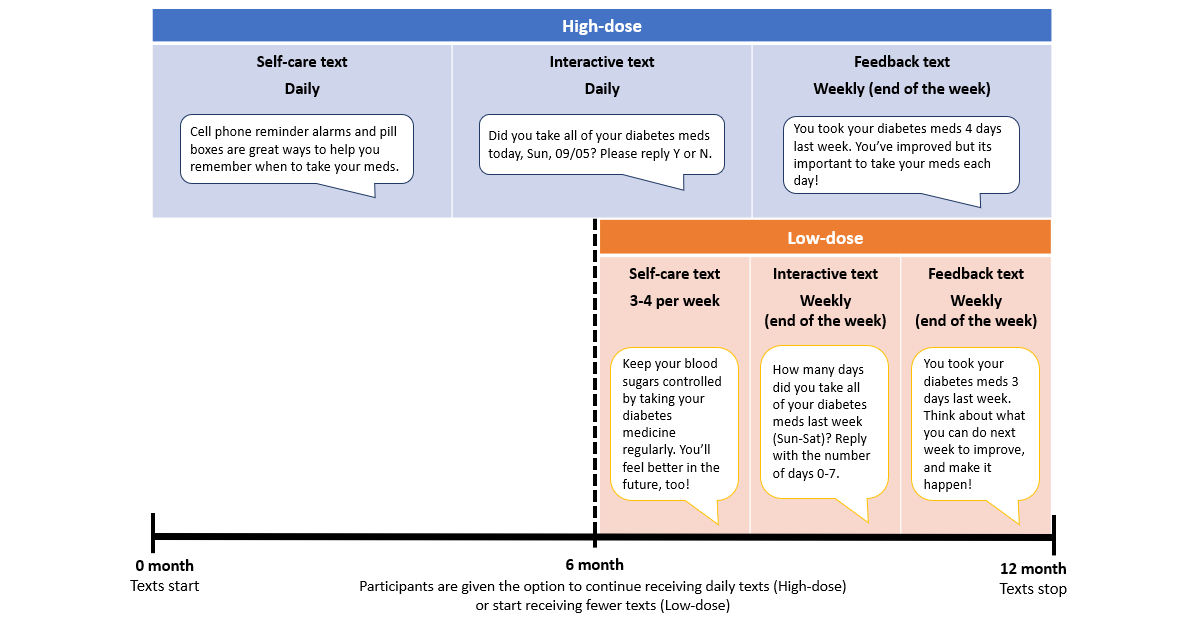
High-dose and low-dose REACH text message frequencies and content examples. REACH: Rapid Education/Encouragement And Communications for Health.

For the first 6 months, all participants received daily self-care texts and daily interactive texts. After 6 months, participants had the option to receive fewer text messages for the remaining 6 months of the intervention (ie, low-dose) or continue to receive daily text messages (ie, high-dose; Figure 1). If we could not reach participants for their 6-month assessment and therefore were not able to present this option, they continued to receive daily text messages (high-dose).

Participants assigned to receive REACH text messages were randomly assigned to also receive monthly phone coaching to set diabetes self-care goals for the first 6 months at a ratio of 1:1 [27,29]. After 6 months, phone coaching ended for those so assigned, all participants were offered the low-dose option described earlier, and all continued to receive REACH text messages for the next 6 months.

### Functionality and Monitoring of Interactive Text Messages

**Figure 2:**
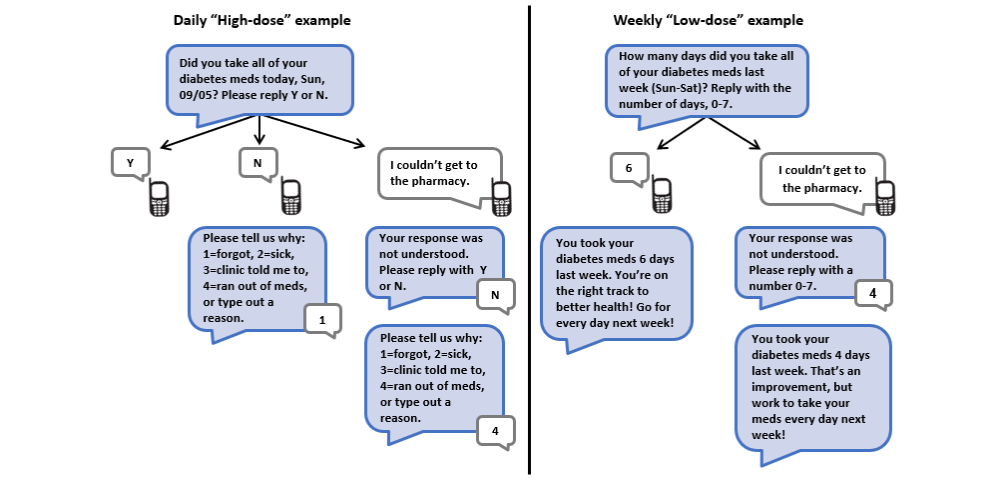
Comparison of functionality for the interactive text message based on whether it was sent daily (high-dose) or weekly (low-dose). Blue messages are intervention texts, and white messages are examples of participant responses.

When sent daily, the interactive text message asked participants if they took all their diabetes medication that day, requesting a yes or no response (Figure 2). On the basis of usability testing results [28], we allowed a range of acceptable response options representing yes and no (eg, yeah, Y, yep, N, and nope). If participants provided a no response, a follow-up text was sent asking why with various response options. Participants received their adherence feedback text based on their daily responses at the end of the week. When sent weekly for participants who chose low-dose, the interactive text asked participants how many days that week they had taken all their diabetes medication, requesting a 0 to 7 response. After participants provided a response, they received their adherence feedback text message (Figure 2).

If the participant responded to the interactive text message with something other than a solicited response, error messages were triggered (Figure 2). The system accepted any response until the next text message was sent the subsequent day.

We monitored participants’ responses to the interactive texts weekly throughout the trial. If a participant did not respond to the interactive message for 2 consecutive weeks, an RA called the participant to identify any technical problems. To avoid pressuring the participants to respond, the RA only asked whether they were experiencing technical problems with receiving or responding to text messages and would troubleshoot as needed. If a participant remained nonresponsive after we confirmed that they were not having problems, we did not make repeat calls.

### Measures

#### Demographics and Clinical Characteristics

We collected self-reported age, gender, race, ethnicity, education (ie, years in school), income, diabetes duration (ie, years since a diabetes diagnosis), and insulin status.

#### Psychosocial Factors

Participants completed self-reported measures of health literacy (Brief Health Literacy Screen [34]), numeracy (Subjective Numeracy Scale, 3-item version [35]), and stressors (Tool for Assessing Patients’ Stressors, 8-item version [36]).

#### Diabetes Self-Care

Participants also completed a self-reported measure of diabetes self-efficacy (Perceived Diabetes Self-Management Scale, 4-item version [37]) and diabetes medication adherence (Adherence to Refills and Medications Scale for Diabetes [38]). In addition, all participants completed an HbA_1c_ test via venipuncture or point of care by the patient's clinic or using a mail-in HbA_1c_ kit provided and analyzed by CoreMedica Laboratories.

#### Engagement

We defined engagement as *any* response to the interactive text messages depicted in Figure 2. If a participant did not respond, it was coded as nonengagement for that day. We calculated text message response rates by dividing each participant’s number of responses by the total number of interactive texts sent to that participant.

To provide a more comprehensive understanding of engagement, beyond average response rate, we assessed response rates over time to explore change in engagement and examined both patient and intervention characteristics associated with response rate to identify factors that may impact engagement. In addition, to understand the more nuanced reasons about why participants chose to engage, we supplemented our objective measure of engagement (ie, response rate) with interview data to inform patients’ subjective experience of their engagement and reasons for their chosen text frequency. This is consistent with the conceptualization of engagement proposed by Perski et al [16].

### Analyses

All statistical analyses were performed using R version 3.5.1. We described patient characteristics using means and SDs or frequencies and percentages as appropriate. We computed the following descriptive statistics regarding text message response rates: means, medians, and the first and third quartiles (interquartiles).

#### Long-Term Engagement

Response rates were examined in 3 ways: during the full 12 months of the intervention, the first 6 months (predose choice), and the last 6 months (postdose choice). There was variation in when participants were offered the low-dose option due to variation in when they completed their 6-month assessment; therefore, we defined the period of prechoice engagement as ≤160 days (the period where no participants had yet been given the choice) and postchoice engagement as ≥240 days (the point at which all participants had made the choice). We excluded the first 30 days to mitigate the impact of participants’ acclimation to the intervention (ie, learning curve).

We used logistic regression to examine whether there was a time trend in the odds of responding during the first 6 months. If participants withdrew during this period, we coded them as having a nonresponse from the day they withdrew through 160 days. We also used logistic regression to examine whether there was a time trend in the last 6 months of the intervention. If participants withdrew after making their choice, we coded them as having a nonresponse from the time they withdrew through 365 days, either daily or weekly, depending on their choice. Both time-trend analyses used generalized estimating equations (GEE) to fit the model under a working exchangeable correlation structure to account for repeated outcome measures [39].

#### Patient Characteristics and Engagement

We used simple linear regression to examine associations between patient characteristics and 6-month response rate and between patient characteristics and change in response rate during the first 6 months. We defined change as each participant’s slope as estimated from a linear fit over time (30 days to 160 days). Patient characteristics included demographics and clinical characteristics (age, gender, race, education, income, diabetes duration, and insulin status), baseline psychosocial factors (health literacy, numeracy, and stressors), baseline diabetes management (diabetes self-efficacy, medication adherence, and HbA_1c_), and clinic site (FQHC vs VUMC). In addition, we used unadjusted odds ratios to examine associations between patient characteristics and dose choice (ie, high-dose or low-dose).

#### Intervention Features and Engagement

To assess whether a human component (ie, monthly phone coaching) enhanced engagement with text messages, we used unadjusted linear regression with the condition (text messages only or text messages plus phone coaching) as the predictor and the 6-month response rate as the outcome. We further used unadjusted logistic regression to examine whether a time trend in the odds of responding during the first 6 months differed based on the assigned condition.

To evaluate whether there was an association between dose choice and postchoice response rate, we used GEE with a working exchangeable correlation structure to account for repeated outcome measures. We used logistic models to examine (1) whether a time trend in the odds of responding during the last 6 months differed based on dose choice, (2) whether there was an association between dose choice and the odds of responding postchoice, and (3) whether there was an association between dose choice and change in the odds of responding from before to after making the choice. Covariates for the latter 2 models included age, gender, and race.

#### Subjective Engagement and Reasons for Dose Choice

We used thematic analysis with NVivo version 11 to identify, organize, and interpret themes in the follow-up interview transcripts [40]. First, we used an inductive approach to construct a codebook based on coders’ preliminary read of the transcripts. The codebook indicated the themes identified from the data. We then applied the initial codebook to a subset of the transcripts to clarify the definitions and resolve discrepancies. All transcripts were then coded independently, with one-third coded by both reviewers to evaluate interrater reliability (ĸ=0.89). We then used deductive analysis on content within relevant themes or structural analysis to answer specific research questions. Specifically, to understand reasons for engagement (responding to texts), we identified subthemes and described content within the themes *medication reminders* and *accountability*. To understand participants’ dose choice, we conducted structural coding of all responses to interview questions about that choice (ie, “After six months in REACH, you chose to receive 3-4 text messages per week/to receive daily text messages for the rest of the program. Why did you choose that option?”).

## Results

### Participants

In the trial, 256 participants were assigned to receive text messages. Of those enrolled, 8 participants were not included in these analyses due to early withdrawal (n=5), a technical error resulting in text messages not being sent (n=2), or opting out of text messages during the first 30 days (n=1). The remaining 248 participants were included in the quantitative analyses. Participants were, on average, aged 55.8 (SD 9.8) years, 55% (137/248) were female, 52% (129/248) were nonwhite, 39% (97/248) were black, 41% (101/248) had a high school degree or less education, and 41% (101/248) had an annual household income of <US $25,000 (Table 1).

Of the 46 participants invited to complete a follow-up interview, 36 (78%) did so. The characteristics of the interviewed participants were similar to those of the larger sample: aged 51.5 (SD 11.0) years, 56% (20/36) female, 67% (24/36) nonwhite, 53% (19/36) black, 44% (16/36) with a high school degree or less education, and 53% (19/36) with an annual household income of <US $25,000.

**Table 1:** Patient characteristics (N=248).

Patient characteristics		Values
Age (years), mean (SD)		55.8 (9.8)
**Gender, n (%)**		
	Male	111 (44.8)
**Race/ethnicity, n (%)**		
	Non-Hispanic White only	119 (48.0)
	Non-Hispanic Black only	97 (39.1)
	Hispanic	15 (6.0)
	Other and multiracial	17 (6.9)
Education (years), mean (SD)		14.1 (3.0)
**Annual household income (US $), n (%)**		
	<25,000	101 (40.7)
	≥25,000	125 (50.4)
	Refused/do not know	22 (8.9)
Diabetes duration (years), mean (SD)		10.9 (7.5)
Insulin status (% prescribed), n (%)		123 (49.6)
Health literacy (BHLS^a^), mean (SD)		13.0 (2.6)
Numeracy (SNS-3^b^), mean (SD)		4.4 (1.3)
Stressors (TAPS-8^c^), mean (SD)		3.8 (2.0)
Diabetes self-efficacy (PDSMS-4^d^), mean (SD)		13.8 (3.5)
Medication adherence (ARMS-D^e^), mean (SD)		39.8 (3.8)
HbA_1c_^f^ (%), mean (SD)		8.6 (1.8)
Clinic site (FQHC^g^), n (%)		103 (41.5)
Assigned condition (text messages plus phone coaching), n (%)		123 (49.6)

^a^BHLS: Brief Health Literacy Scale; Possible score range: (3-15).

^b^SNS-3: Subjective Numeracy Scale; Possible score range: (1-6).

^c^TAPS-8: Tool for Assessing Patient Stressors; Possible score range: (0-8).

^d^PDSMS: Perceived Diabetes Self-Management Scale; Possible score range: (4-20).

^e^ARMS-D: Adherence to Refills and Medications Scale for Diabetes; Possible score range: (11-44), items are reserve-scored so higher scores indicate greater adherence.

^f^HbA_1c_: hemoglobin A_1c_.

^g^FQHC: federally qualified health center.

### Long-Term Engagement

**Figure 3:**
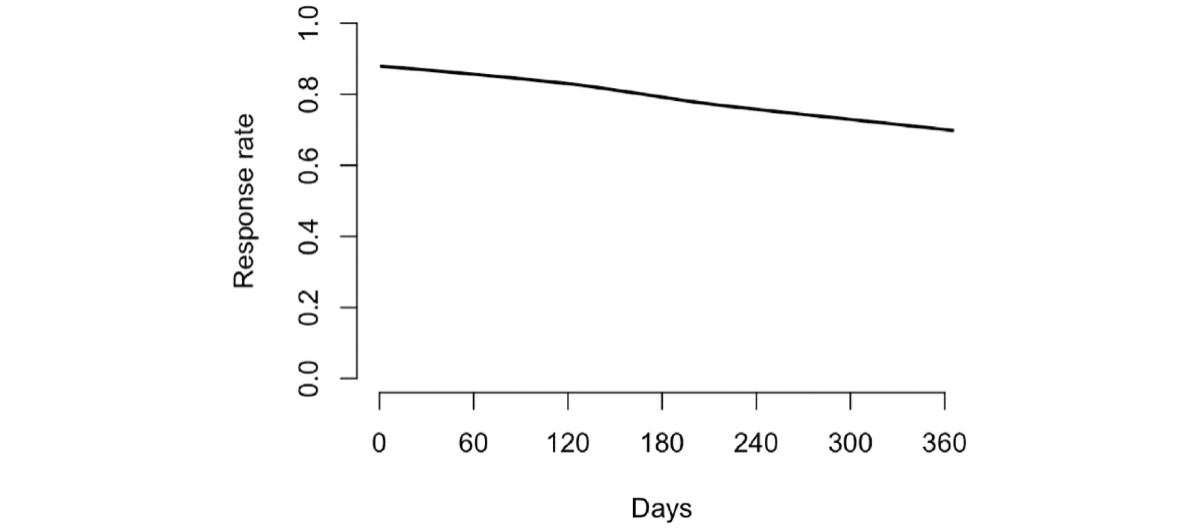
Predicted response rates over the 12-month intervention using a locally weighted scatterplot smoothing (LOWESS) curve.

Participants responded to a mean of 81% (SD 23%; median 91%; IQR 75%-97%) of the interactive text messages over the 12-month intervention. Less than 1% of participants’ responses to these texts were unexpected responses (ie, a response other than yes or no to the daily text message or 0 through 7 to the weekly text message). Response rates were negatively skewed such that over two-thirds of participants’ response rates were greater than 75%. Participants’ average response rate decreased over time, although the predicted rate never dropped below 70% (Figure 3).

We also examined engagement separately during the first and last 6 months of the intervention. During the first 6 months, participants responded to a mean of 85% (SD 20%; median 94%; IQR 80%-98%) of the interactive text messages, and during the last 6 months, participants responded to a mean of 81% (SD 22%; median 92%; IQR 75%-97%). For both time periods, we found statistically significant time trends such that participants had decreased odds of responding over time, although there was little substantive change (Table 2). We estimated the odds of responding to be about 0.7% lower on a given day as compared with the previous day during the first 6 months and to be about 0.4% lower on a day-by-day basis during the latter 6 months (Table 2).

**Table 2:** Results for logistic regression models examining time trends in responding.

Time trends and dose choice associations			Estimate		95% CI		*P* value
**Time trends in response rates before dose choice (first 6 months)**							
	Day	0.993		0.988-0.998		.008^a^	
	Condition (text messages plus phone coaching)^b^	0.840		0.527-1.337		.46	
	Day×condition (text messages plus phone coaching)	1.002		0.999-1.006		.09	
**Time trends in response rates after dose choice (latter 6 months)**							
	Day	0.996		0.995-0.998		<.001^a^	
	Low-dose choice^c^	0.613		0.364-1.032		.07	
	Low-dose choice×day	1.001		0.999-1.002		.16	
**Association between dose choice and (subsequent) response rate^d^**							
	Low-dose choice	0.842		0.612-1.157		.29	
**Association between dose choice and (subsequent) change in response rate^d^**							
	Low-dose choice	0.745		0.538-1.032		.08	
	Postchoice responding	0.439		0.334-0.579		<.001^a^	
	Low-dose choice×postchoice responding	1.208		0.931-1.567		.16	

^a^Significant based on *P*<.05.

^b^Reference group is texts only.

^c^Reference group is choosing high-dose (high-dose choice).

^d^Covariates included age, gender, and race.

### Participant Characteristics and Engagement

Patients’ clinic site, age, gender, education, income, diabetes duration, insulin status, health literacy, numeracy, and diabetes self-efficacy were not associated with 6-month engagement (Table 3). However, black race (compared with white), worse baseline medication adherence and worse HbA_1c_ were associated with lower engagement, although the effects were small (2%-7% lower; Table 3). We did not find evidence of an association between any measured patient characteristics and change in 6-month engagement, and we did not find evidence of an association between any measured patient characteristics (including prechoice response rate) and dose choice (Table 3).

**Table 3:** Associations between patient characteristics and engagement.

Patient characteristics		6-month response rate, estimate (95% CI)	Change in 6-month response rate, estimate (95% CI)	Low-dose choice, OR^a^ (95% CI)
Age (years)		–0.001 (–0.003 to 0.003)	–0.001 (–0.004 to 0.002)	0.989 (0.961-1.017)
**Gender**				
	Male	–0.015 (–0.065 to 0.039)	0.030 (–0.027 to 0.085)	0.733 (0.406-1.295)
**Race/ethnicity^b^**				
	Non-Hispanic Black vs non-Hispanic White	–0.070^c^ (–0.129 to –0.012)	–0.013 (–0.073 to 0.049)	0.845 (0.432-1.545)
Education (years)		0.002 (–0.008 to 0.013)	0.002 (–0.008 to 0.012)	1.074 (0.980-1.189)
Annual household income ≥US $25,000		0.040 (–0.013 to 0.098)	0.021 (–0.100 to 0.187)	1.055 (0.600-1.881)
Diabetes duration (years)		–0.001 (–0.004 to 0.002)	0.001 (–0.003 to 0.004)	0.989 (0.951-1.028)
Prescribed insulin		–0.040 (–0.092 to 0.013)	–0.015 (–0.069 to 0.041)	0.808 (0.451-1.417)
Health literacy (BHLS^d^)		–0.000 (–0.010 to 0.009)	–0.000 (–0.013 to 0.013)	0.994 (0.890-1.123)
Numeracy (SNS-3^e^)		–0.001 (–0.023 to 0.021)	0.002 (–0.021 to 0.026)	1.055 (0.841-1.322)
Stressors (TAPS-8^f^)		–0.006 (–0.019 to 0.007)	–0.008 (–0.022 to 0.004)	0.945 (0.821-1.080)
Diabetes self-efficacy (PDSMS-4^g^)		0.008 (–0.000 to 0.016)	0.004 (–0.003 to 0.011)	1.004 (0.924-1.096)
Medication adherence (ARMS-D^h^)		0.014^c^ (0.006 to 0.023)	0.005 (–0.003 to 0.013)	0.997 (0.925-1.084)
HbA_1c_^i^ (%)		–0.019^c^ (–0.036 to –0.004)	0.001 (–0.015 to 0.018)	0.869 (0.728-1.006)
Clinic site (FQHC^j^)		0.005 (–0.045 to 0.058)	–0.016 (–0.072 to 0.038)	0.680 (0.378-1.188)
Assigned condition (text messages plus phone coaching)		0.008 (–0.044 to 0.060)	0.024 (–0.031 to 0.081)	1.438 (0.847-2.583)
Prechoice response rate		N/A^k^	N/A	1.247 (0.262-7.288)

^a^OR: odds ratio.

^b^Due to the small number of participants who identified as either Hispanic or multiracial, we did not report associations for these groups.

^c^Significant association based on 95% CI.

^d^BHLS: Brief Health Literacy Scale.

^e^SNS: Subjective Numeracy Scale.

^f^TAPS-8: Tool for Assessing Patient Stressors.

^g^PDSMS: Perceived Diabetes Self-Management Scale.

^h^ARMS-D: Adherence to Refills and Medications Scale for Diabetes.

^i^HbA_1c_: hemoglobin A_1c_.

^j^FQHC: federally qualified health center.

^k^N/A: not applicable.

### Intervention Features and Engagement

Participants’ assigned condition (ie, whether they received text messages only or text messages plus phone coaching) was not associated with their 6-month response rate (Table 2). In addition, the odds of responding over time did not differ by condition (Table 2).

Among participants given the option to receive fewer text messages at 6 months (n=213), 55.9% (119/213) of participants chose to receive fewer texts and 44% (94/213) chose to continue receiving daily texts. The remaining 35 participants were not asked this question because of not completing the 6-month assessment, being unreachable by phone, or an RA error. Dose choice was not associated with the odds of responding postchoice (Table 2). When we examined whether dose choice was associated with a change in the odds of response, from before to after making the choice, there was no association. Odds of responding were lower over time in both groups but did not differ by choice (Table 2).

### Subjective Engagement

#### Reasons for Engaging With Text Messages

The most common theme about why participants responded to the interactive text is because they used it as a reminder for taking their medicine (n=19). For instance, participants described how receiving and responding to the text served to routinize their medication taking and helped increase their awareness:

My little reminder I received on the cell phone, the text, would, you know, help me remember to take my medicine. Helped me get on a regular schedule.

Hispanic white male, aged 45 years

Participants explained how they came to anticipate the text messages which helped them remember to take their medications before they received the message. Sometimes, the text message was a cue to action if they had not yet taken their medication:

I was forgetting to take my medications. Once I started getting the messages...I would go get it and take it. And it would remind me...I knew it was coming, because I picked the evening hours to make sure that, at eleven o'clock, I would get the message...If I didn't, I know I had enough time to still take it for that day.

Hispanic white female, aged 50 years

The other, less common theme, explaining participants’ responses to the interactive text messages, was feeling accountable—either to themselves or to the REACH team (n=9). Some participants specifically described how they looked forward to being able to respond *yes* to the text message when they had taken their medication, whereas others talked about negative feelings if they had not taken their medication and not wanting to respond with a *no*:

Each day I got a message. If I didn't take my medicine, I was taking it. And if I did take my medicine, I was waiting on that message to feel like I did something right.

Non-Hispanic black female, aged 26 years

So, it was like when I didn't want to respond, it was because I hadn't taken my medicine. Then, I felt guilty and I felt that I was cheating myself.

Non-Hispanic black female, aged 31 years

In contrast, some described the importance of being honest in their responses and not wanting to lie if they had not taken all their diabetes medications that day. Finally, a few participants described feeling like someone was keeping tabs on them, one referring to us as “big brother”:

It kept you on track, gave you reminders, and it was like you having like this little voice in the phone telling you, “Make sure you take your medicine,” and, you know, do what you're supposed to do. Stay on track.

Black female, ethnicity not reported, aged 60 years

#### Reasons for Dose Choice

Of the 36 participants who completed a follow-up interview, 64% (23/36) chose the low-dose option, 25% (9/36) chose to keep the high-dose option, and 11% (4/36) were not asked because of the reasons listed earlier. Common themes in participants’ reasons for choosing the low-dose option included that they had established a routine (n=6), the text messages were interfering with their schedule (n=4), and they were becoming frustrated with the text messages (n=6). Participants who mentioned having a routine shared how the text messages had helped them to create a habit, and therefore, they felt they no longer needed the text messages as often:

I think I had reached the point where the text messages had the desired effect on me, and so I was sort of trained at that point to anticipate them and to be aware of their purpose...so fewer text messages still had the same purpose.

Non-Hispanic white male, aged 60 years

Some participants who switched to low-dose felt that their schedules were too busy to attend to and keep up with the messages on a daily basis, whereas others shared how they were frustrated with the text messages either because the content felt redundant or because they did not like how many texts were being sent:

I find you’re getting too much. It’s too much. It’s too much. And a reminder here or there is fine. But to be getting constant messages like that...I personally find it annoying.

Non-Hispanic black female, aged 60 years

Less commonly mentioned reasons included wanting more autonomy to manage diabetes without the assistance of the text messages (n=2) and issues pertaining to their cell phone or plan (n=3; eg, limited plans made it difficult to receive daily text messages and still be able to send text messages toward the end of the month).

The most common reason participants provided for wanting to continue receiving high-dose was the helpfulness of the daily text messages (n=9). Specifically, participants stated that the text messages benefited their health, and they wanted to continue receiving them daily to keep experiencing that benefit. Many mentioned how the interactive texts made them feel more engaged in their diabetes self-care and reminded them to take their medications each day:

*I’m very forgetful...It’s going to sound silly, but* [it’s] *sort of like a security blanket. You know, by receiving that message, it’s like, okay, I felt like I was more involved in taking care of my diabetes. I felt like I was participating more in something.*

Non-Hispanic white male, aged 45 years

If for some reason I hadn’t taken my medication that day, because I got busy doing other things, when a message comes in reminding me, [I think] “Oh, I haven’t taken the medication.” So I will go ahead and take it, then answer them. If that were to happen every other two days or whatever, then maybe I would have missed taking my medication...It brought you back, even when you’re on a really busy day. You’re able to get that message and say, “Oh yeah” and take your medicine.

Hispanic female, aged 65 years

Other participants specifically mentioned the self-care promotion text messages and appreciated the tips and information included in that content. Finally, some emphasized improvements in their health, which they attributed to the daily text messages:

I guess, you know, texting is a part of life now...it’s just kind of a daily thing, and it’s information I can share with somebody else. “Oh, look what I got.” Or if you’re somewhere all you have to do is show your phone...I just liked the daily input, and it was good information.

Non-Hispanic black female, aged 47 years

When I noticed my A1c had went down, I believe in that period it was just like, “Oh, it worked.” So, I think this is what made me [say] “Nah, I need it because some progress is better than none.”

Non-Hispanic black female, aged 31 years

## Discussion

### Principal Findings

We examined user engagement in a 12-month text messaging intervention designed to support diabetes self-care in a sample overrepresenting adults with low SES and racial/ethnic minorities. We wanted to understand how intervention characteristics and patient characteristics affected engagement with the text messages, with particular attention to the addition of a human component and participants’ opportunity to choose their text message frequency after 6 months of daily texts. Engagement gradually decreased throughout the intervention but remained relatively high over 12 months. We did not find any differences in engagement by participants’ clinic site, age, gender, education, income, diabetes duration, insulin status, health literacy, or numeracy. Engagement was lower among participants who were black and who had worse medication adherence and HbA_1c_ at baseline; however, the magnitude of these differences was relatively small. When given the option at 6 months to receive fewer text messages, nearly half of the participants chose to continue receiving daily text messages for the remaining 6 months. We did not find differences in engagement based on participants’ receipt of phone coaching or their selected text message frequency.

The literature reporting on engagement with mobile health (mHealth) interventions is growing, although few studies report on interventions lasting more than 6 months [17]. Our findings support the use of text messages to engage participants long term. Before starting the RCT, we conducted iterative usability testing with participants from the target population; we addressed participant concerns regarding content and functionality, which may have helped sustain engagement in the trial [26,28]. In addition, the personalization used in REACH (eg, tailored content and message timing) may have promoted engagement, which is consistent with other studies employing individualized content [12]. Black participants and those with worse baseline medication adherence and HbA_1c_ had lower engagement. Notably, although these differences were statistically significant, average engagement was still relatively high across all participants (eg, the average response rate among black participants was 80%, whereas for white participants, it was 87%). On the basis of the qualitative data, participants’ engagement appeared to be influenced by the perceived helpfulness of the text messages with reminding them to take their medication and feelings of accountability engendered by the texts.

Studies exploring the role of human support in engagement with automated technologies have shown inconsistent findings [26,41]. Variation in results are likely due to variation in the type of human involvement (eg, remote vs in person, familiar vs new health care professional, and providing structured support vs following up as issues arise), the type of technology, the engagement measure, and users’ needs and preferences [41]. In this study, there was no difference in text message response rates between users who received text messages only and those who received both text messages and monthly phone coaching. Mohr et al [42] proposed a model for how human support increases adherence to electronic health interventions, such that patients experience accountability to a person they view as trustworthy, benevolent, and having expertise. Although the text messages in this study were automated, the content contained encouragement, information, and bidirectional communication, which may have been perceived as human support, thereby increasing engagement, regardless of assignment to phone coaching. In a study that explored perceptions of a similar automated text messaging program for diabetes self-care, black patients with T2D shared how they felt high levels of social support and cared for by a person or friend despite knowing that the program was automated [43]. Furthermore, in this study, all participants experienced a human component from interacting with the study staff for other elements of the study (eg, completing surveys), which may have dampened our ability to detect additive effects of the monthly coaching.

The ideal dose or frequency of text messages to sustain engagement in an intervention is unclear and likely varies by individual user. We did not find any patient characteristics associated with frequency choice, which suggests that preference was idiosyncratic. We included the low-dose option to sustain engagement among participants who may prefer receiving text messages less often than daily. The variability in choice and the specific reasons for making the choice suggest it was valuable to participants. The choice may also have kept engagement high for some participants who otherwise would have stopped responding. Reconciling dose preference with the dose needed to impact patient outcomes will be integral for the design of future text messaging interventions [13].

### Limitations

This study recruited patients with T2D from a specific region in middle Tennessee. Therefore, we acknowledge that the findings may not be generalizable to patients with T2D in other locations or to other patient populations. In addition, because patients who did not want to respond to text messages were likely those who chose not to participate in this study, this may have led to a self-selection bias among our sample. As mentioned earlier, interactions with study staff may have reduced our ability to detect the effects of human coaching on engagement. We did not find evidence that patient characteristics were associated with dose choice; however, it is possible that characteristics not assessed in this study may have impacted the decision. For example, in the case of employment status, a person who is retired may be more receptive to receiving a higher frequency of messages than someone who is working full time. Finally, we were unable to compare how engagement would have been impacted if we did not give a choice for frequency preference.

### Conclusions

There is growing evidence for the efficacy of text message–delivered interventions to improve adherence and clinical outcomes [11], and this study addresses important gaps by examining long-term engagement and associations between engagement and participant and intervention characteristics. As the literature on engagement is growing, there have been calls for more standardized engagement reporting to compare results across studies and draw more concrete conclusions about the role of engagement [44,45]. Kelders and Kip [46] recently developed a self-report scale to capture multiple components of engagement with health technologies (ie, behavior, cognition, and affect). Similarly, we encourage reporting on usage that goes beyond the average engagement level, but rather examines how engagement changes over the course of an intervention, and factors that influence engagement. In any intervention, users will become fatigued over time, so testing engagement promotion strategies (including new content to sustain interest over time and/or gamification) will also provide insights on how to improve and sustain engagement [47,48]. Finally, robust measurement of engagement will also help us explore the effect of engagement on outcomes to enhance our understanding of how and for whom mHealth interventions improve health. As a next step to this study, we plan to evaluate how engagement with REACH impacts outcomes, including HbA_1c_ and medication adherence. Specifically, we will explore whether there is a minimum engagement threshold (ie, response rate) needed for patients to experience improvements in outcomes.

As patients with low SES, who are less likely to have smartphones and internet access, also tend to have worse health outcomes, the spread of apps and other internet-dependent technologies may unintentionally widen health disparities [49]. Compared with other forms of mHealth technology, text messages offer key advantages given their ubiquity and potential for scalability [12,50]. This study contributes to evidence for the case that these interventions may help create equity if implemented in clinical care.

## Abbreviations

FQHC: federally qualified health center
GEE: generalized estimating equations
HbA1c: hemoglobin A1c
mHealth: mobile health
RA: research assistant
RCT: randomized controlled trial
REACH: Rapid Education/Encouragement And Communications for Health
REDCap: Research Electronic Data Capture
SES: socioeconomic status
T2D: type 2 diabetes
VUMC: Vanderbilt University Medical Center
